# Response Activation and Inhibition in Patients With Prolactinomas: An Electrophysiological Study

**DOI:** 10.3389/fnhum.2020.00170

**Published:** 2020-07-23

**Authors:** Jian Song, Chenglong Cao, Yu Wang, Shun Yao, Michael P. Catalino, Deqi Yan, Guozheng Xu, Lianting Ma

**Affiliations:** ^1^Department of Neurosurgery, The General Hospital of Chinese People’s Liberation Army Central Theater Command, Wuhan, China; ^2^The First School of Clinical Medicine, Southern Medical University, Guangzhou, China; ^3^Key Laboratory of Cognitive Science, College of Biomedical Engineering, South- Central University for Nationalities, Wuhan, China; ^4^Center for Pituitary Tumor Surgery, Department of Neurosurgery, The First Affiliated Hospital, Sun Yat-sen University, Guangzhou, China; ^5^Department of Neurosurgery, Brigham and Women’s Hospital, Harvard Medical School, Boston, MA, United States; ^6^Department of Neurosurgery, University of North Carolina, Chapel Hill, NC, United States; ^7^Traditional Chinese Medicine College, Xinjiang Medical University, Urumqi, China

**Keywords:** prolactinoma, ERPs, response activation, response inhibition, P300

## Abstract

Impairment of executive function has been reported in patients with prolactinomas. However, few studies have investigated the electrophysiological mechanisms of response activation and response inhibition in these patients. In this study, we employ an event-related potentials (ERPs) technique to quantitatively assess response activation and inhibition before and after the surgical treatment of prolactinomas. A 64-electrode electroencephalogram (EEG) skullcap was used to record the brain activity in 20 pre-operative patients, 20 follow-up post-operative patients, and 20 healthy controls (HCs) while performing the visual Go/Nogo task. As expected, we identified P300 across all study populations that could reflect response activation and inhibition. Across the three groups, the Nogo stimuli evoked larger frontal-central P300 than the Go stimuli did. In contrast, the Go trials elicited larger parietal P300 than the Nogo trials did. The peak latency of P300 was significantly delayed in both the pre-operative and the post-operative groups compared to the HCs. The amplitude of P300 in both the Go and the Nogo conditions was significantly decreased in the pre-operative patients compared with that of the HCs. At 6 months post-operatively, the prolactinoma patients showed an increase in amplitude of P300 during both the Go and the Nogo tasks. These findings indicate that the prolactinoma patients suffer from deficits in response activation and inhibition, which could be improved by surgical treatment.

## Introduction

Pituitary tumors are the second most common intracranial tumors, accounting for about 16.5% of central nervous system tumors (Ostrom et al., 2018). Among all subtypes of pituitary tumors, prolactinomas, which are characterized by an overproduction of prolactin (PRL), are the most common (Fleseriu et al., 2006). Beyond the clinical symptoms caused by pituitary diseases (Pal et al., 2019), there are emerging reports on cognitive impairments and emotional disorders (Pertichetti et al., 2019). Substantial evidence has shown that patients with pituitary tumors frequently report deficits in attention (Bala et al., 2016), working memory (Brummelman et al., 2011), executive function (Yao et al., 2017), depression (Meyers, 1998), and emotion processing (Song et al., 2018).

Abnormal hormone levels can be associated with cognitive impairments (Labad, 2019). Our team has conducted a retrospective study on the relationship between cognitive function and endogenous PRL levels, showing that higher PRL levels were associated with worse executive function and verbal memory (Yao et al., 2017). However, there is no concrete evidence supporting the altered response activation and inhibition in prolactinoma patients. Elevated PRL levels have shown adverse morphological effects on specific brain structures (Yao et al., 2017), functional connectivity (Yao et al., 2020c), and cognitive function (Bala et al., 2016; Yao et al., 2017). Prolactin also suppresses estrogen and testosterone, which impacts synaptogenesis and axonal guidance and, subsequently, influences neuron numbers and morphology (Simerly, 2002). Generally, cognition involves the set of processes that mediate between stimulus and response. Executive dysfunction is often related to negative inhibitory processing and can persist after the rehabilitation of affective disorders (Barkley, 2001). Neurological imaging studies have reported that the inhibition mechanism requires the activation of the frontal lobe and other brain regions, such as the prefrontal cortex (PFC) and the anterior cingulated cortex (ACC; Rubia et al., 2001). The inhibitory control functions of the frontal cortex are responsible for inhibiting unrelated thoughts or actions towards specific tasks, self-monitoring, and self-regulating (Kuwabara et al., 2014). Furthermore, although these inhibitory functions substantially rely upon the frontal cortex, they may also depend upon both incoming and outgoing pathways involving many other cortical and subcortical structures (Rubia et al., 2001).

Event-related potentials (ERPs) are real-time measures of neural activity with high temporal resolution. An ERP technique has been used to study the electrophysiological mechanisms of cognitive processing (Sutton et al., 1965). Importantly, the Go/Nogo paradigm has been widely adopted to assess inhibitory control (Yang et al., 2009). However, no studies have evaluated response activation and inhibition in prolactinoma patients using ERPs with the Go/Nogo paradigm. One major ERP component elicited during the Go/Nogo paradigm is P300. The amplitude of P300 reflects the quantity of neural resources engaged in processing task-relevant stimuli (Polich and Kok, 1995). The P300 component elicited by the Go/Nogo paradigm has been correlated with behavioral success while performing executive tasks (Downes et al., 2017; Gao et al., 2017), and response inhibition (in the Nogo condition) is more cognitively demanding than response activation (in the Go condition; Gao et al., 2017). P300nogo is highly relevant during conflict inhibition, an enhanced positive peak at the anterior electrodes in Nogo relative to Go condition with a duration of 300–500 ms after stimulus onset (Polich and Kok, 1995; Bokura et al., 2001; Polich, 2007). Topographically, the amplitude of P300nogo is maximal anteriorly, whereas that of P300go is maximal posteriorly (Kopp et al., 1996; Fallgatter and Strik, 1999). A preponderance of evidence focuses on inhibitory processing since it critically engages important cognitive functions, such as suppressing inappropriate actions, attentional control, and working memory (Aron et al., 2014; Erika-Florence et al., 2014). A few studies simultaneously measured response activation and inhibition in patients with psychiatric disorders. The findings suggest that the amplitude of P300 was reduced within the anterior regions in the Nogo condition in patients with schizophrenia compared with healthy controls (HCs; Fallgatter et al., 2003). Kleinlogel et al. (2007) found that patients with schizophrenia showed P300 reductions in Nogo and Go conditions and an increase in P300nogo latency. These studies suggest that reduced P300nogo and P300go might indicate a dysfunction of response activation and inhibition in patients with schizophrenia. Interestingly, some have proposed the use of psychophysiological measurements, including the P300 component, to be a biomarker for addictions (Fu et al., 2008; Kamarajan et al., 2015). However, to our knowledge, the P300 component has not been used to investigate response activation and inhibition in prolactinoma patients. Our previous findings have shown impairment at the earlier stage of inhibitory control processing in prolactinoma patients (Cao et al., 2017); However, no studies have been conducted to investigate both response activation and inhibition in patients with prolactinomas before and after surgical treatment.

Thus, in the current study, our primary aims were to: (1) determine the electrophysiological markers of response activation and inhibition impairment in prolactinomas; and (2) compare their performance pre- and post-operatively. We hypothesized that pre-operative patients may show a decrease in response activation and inhibition and that, after 6 months, post-operative patients may have improved performance in response activation and inhibition in both the Go and the Nogo conditions. Moreover, the amplitude of P300nogo should be larger than that of P300go in fronto-parietal areas. In contrast, the amplitude of P300go should be larger than that of P300nogo in the parietal area.

## Materials and Methods

### Participants

Twenty pre-operative prolactinoma patients, 20 post-operative prolactinoma patients, and 20 HCs voluntarily participated in this study. All the patients were identified through the Department of Neurosurgery, Wuhan School of Clinical Medicine, Southern Medical University (China) during diagnostic hospitalization. Patients were included if: (1) they were diagnosed with a prolactin-secreting pituitary tumor (Melmed et al., 2011) and were intolerant and/or resistant to long-term drug treatment; (2) had undergone surgery by a transsphenoidal approach and, after 6 months post-operatively, serum prolactin was within the normal range (PRL: 2.64–13.13 ng/ml for males and 3.34–26.72 ng/ml for females); (3) had no history of craniotomy or radiation therapy; and (4) could complete ERP tests. Patients were excluded if they: (1) had a history of neurologic or psychiatric disorders; (2) had comorbidities that could affect cognitive function, including severe liver, heart, or kidney dysfunction; (3) had severe complications, such as coma, infection, epilepsy, hydrocephalus, and leaking of cerebrospinal fluid; and (4) had drug or alcohol abuse [subjects who drink alcohol over 2.0 standard drinks (10 g of pure alcohol) during the day and meet any two of the 11 criteria under DSM-V in the past year; American Psychiatric Association, 2013] or were on any medications (including oral contraceptives). All the prolactinoma patients took part in the ERP test on the first day after their admission to the hospital. They routinely underwent complete ophthalmologic examinations at admission, including the E chart test, which was used to measure best-corrected visual acuity. The participants who could not perform ERP tests due to a visual defect were excluded. The HCs were recruited from healthy volunteers who were matched with age, gender, and education (Table 1). The study was approved by the ethics committee of Wuhan School of Clinical Medicine, Southern Medical University. The study procedures were explained carefully, and informed consent was obtained from all the participants.

**Table 1:** Demographic and clinical characteristics: pre- and post-surgery patients, and healthy controls (HCs).

	Pre-surgery patients (*n* = 20)	Post-surgery patients (*n* = 20)	HCs (*n* = 20)	*p*-Value
Age (years)	34.75 (30.00–40.00)	34.75 (30.00–40.00)	33.95 (29.00–38.00)	0.537^a^
Gender: Male	10 (50%)	10 (50%)	8 (40%)	*χ*^2^ = 0.536
Female	10 (50%)	10 (50%)	12 (60%)	0.765^b^
Education (years)	12.35 (9.00–16.00)	12.35 (9.00–16.00)	12.70 (9.00–16.00)	0.706^a^
Serum PRL (ng/ml)	135.44 (28.50–270.00)	12.05 (4.65–19.42)	NA	
Serum E2 (pg/ml)	35.15 (7.00–79.61)	25 (4.25–65.67)	NA	
Serum progesterone (ng/ml)	1.17 (0.12–2.41)	1.12 (0.22–3.59)	NA	
Serum FSH (mIU/ml)	6.27 (2.61–21.00)	7.04 (2.16–20.5)	NA	
Serum LH (mIU/ml)	4.61 (1.89–8.45)	3.65 (1.85–14.34)	NA	
Serum testosterone (ng/ml)	1.04 (0.12–4.47)	1.47 (0.07–8.27)	NA	
Serum GH (ng/ml)	1.078 (0.091–3.144)	0.69 (0.05–2.35)	NA	
Serum TSH (mIU/ml)	2.135 (0.96–3.91)	2.02 (0.765–3.80)	NA	
Serum cortisol (nmol/l)	309.45 (188.10–446.90)	289.35 (172.50–355)	NA	

*Data are presented as mean values and ranges (minimum and maximum values). ^a^One-way analysis of variance (ANOVA), ^b^Chi-Square Tests. PRL, prolactin; E2, estradiol; FSH, follicle-stimulating hormone; LH, luteinizing hormone; GH, growth hormone; TSH, thyroid-stimulating hormone*.

### Endocrinological and Radiological Assessment

Endocrinological and radiological study procedures have been described in our previous study (Yao et al., 2017, 2020c). After admission, endogenous hormone levels were measured between 8:00 and 10:00 a.m. to overcome the circadian variation. On the same day, all the prolactinoma patients had a brain MRI performed pre- and post-operatively. Blood samples and additional MRI scans were also obtained from the 20 patients at 6 months after surgery. The brain MRI scans all showed complete tumor resection and no compression to adjacent tissues as assessed by two senior neurosurgeons who were blinded to the patients’ clinical features. None of the HCs showed structural brain abnormalities.

### Stimuli and Procedure

**Figure 1:**
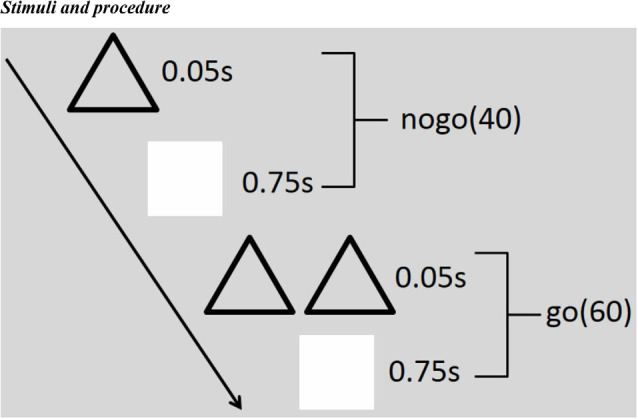
Illustration of the stimulus paradigm applied. There are three blocks in total, and each block includes 60 Go and 40 Nogo stimuli. Each stimulus emerged for 50 ms, followed by an inter-stimulus interval (750 ms). As soon as the Go (double triangles) stimuli appeared onto the screen, the subjects should press the button quickly but should not press the button when the Nogo (single triangles) stimuli turned up.

Single triangles (Nogo) or double triangles (Go) were used as visual stimuli. The stimulus appeared in the central computer screen (light degree = 60 cd/m^2^). The participants sit in a semi-dark test room, with the screen 100 cm away from their eyes and 4° × 4° visual angle. Sixty Go and 40 Nogo stimuli constituted one block, and there were three blocks in total. As soon as the Go stimuli appeared onto the screen, the subjects should press the button quickly but should not press the button when the Nogo stimuli turned up. Each stimulus was presented for 50 ms, followed by an inter-stimulus interval (750 ms; see Figure 1). Before the formal electroencephalogram (EEG) recording, the participants made a full practice to make sure that their performance was representative. During the whole process, the participants needed to watch the screen center leisurely and reduce their eye blinks and body movements as much as possible.

### EEG Recording

Raw EEG was recorded using 64 electrodes with the international 10-10 system (eego™, sampling rate: 1,000 Hz), and all electrodes impedances were kept lower than 5 kΩ.

### EEG Analysis

EEGLAB toolbox, an open-source toolbox running in the MATLAB environment, was used to preprocess the raw EEG (Delorme and Makeig, 2004). The EEG signals to the scalp were continuous at the frontal, central, and parietal electrode sites. Continuous EEG data were re-referenced off-line to the average of both mastoids (M1 and M2) and filtered with a 1–49-Hz bandpass filter. The blink artifact was corrected by hand using SASICA (a plugin from EEGLAB; Chaumon et al., 2015). Epoch was extracted by a time window from 200 ms pre-stimulus to 800 ms post-stimulus and was baseline-corrected based on the pre-stimulus interval.

The stimulus-locked ERPs were averaged over epochs for each participant. P200, N200, and P300 were extracted by inspecting the grand averaged stimulus-locked ERPs for all participants. P300 evaluates response activation and inhibition; hence, this research focused mainly on the P300 component. The time window for capturing P300 is 350–500 ms. Furthermore, the ERP properties, such as the latency of P300, the amplitude of P300, and the average amplitude of P300, were extracted by using Darbeliai (a plugin from EEGLAB).

### Statistical Analysis

The measurements of reaction time and accuracy rate were subjected to one-way analysis of variance (ANOVA) between groups (pre-operative group, post-operative group, and HCs) after homogeneity and normality test of variance. *Post hoc* least significant difference (LSD) test was performed to analyze the significant effects shown by the ANOVA.

After the homogeneity and normality test of variance, three (groups: pre-operative, post-operative, and HCs) × two (stimulus types: Nogo and Go trials) ANOVA was performed on the latency of P300, the amplitude of P300, and the average amplitude of P300 at the regions of interest (ROIs; F1, F2, C1, C2, P1, and P2). *Post hoc* LSD test was performed to analyze the significance of the effects shown by the ANOVA. We investigated Pearson’s correlations between PRL levels and P300 amplitudes in both the Go and the Nogo conditions in pre-operative patients, with a significance level set at *p* < 0.05 (one-tailed and Bonferroni-corrected).

## Result

### Demographic and Clinical Characteristics

The demographic and clinical characteristics of all the participants are presented in Table 1. Most participants, regardless of group, were women. Additionally, there were no differences in age (*p* = 0.537) or education (*p* = 0.706) between the groups.

### Reaction Time and Accuracy Rate

**Figure 2:**
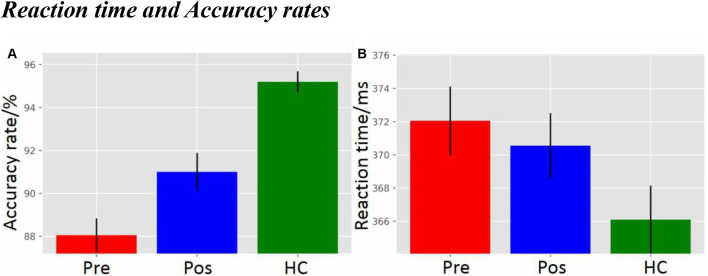
**(A)** Accuracy rate, and **(B)** Reaction time. pre, pre-operative group; post, post-operative group; HC, healthy control group.

As displayed in Figure 2A, there was a significant difference in accuracy rate between groups (*F*_(2,59)_ = 22.78, *p* = 5.3692e-08). The *post hoc* LSD test indicated that the pre-operative accuracy rate was significantly lower than that of the post-operative group (*p* = 0.0075) and the HCs (*p* = 9.4657e-09). The post-operative accuracy rate was significantly lower than that of the HCs (*p* = 2.2132e-04). There was, however, no difference in main group effect for reaction time (Figure 2B; *F*_(2,59)_ = 2.21, *p* = 0.1193).

### Event-Related Potentials

**Figure 3:**
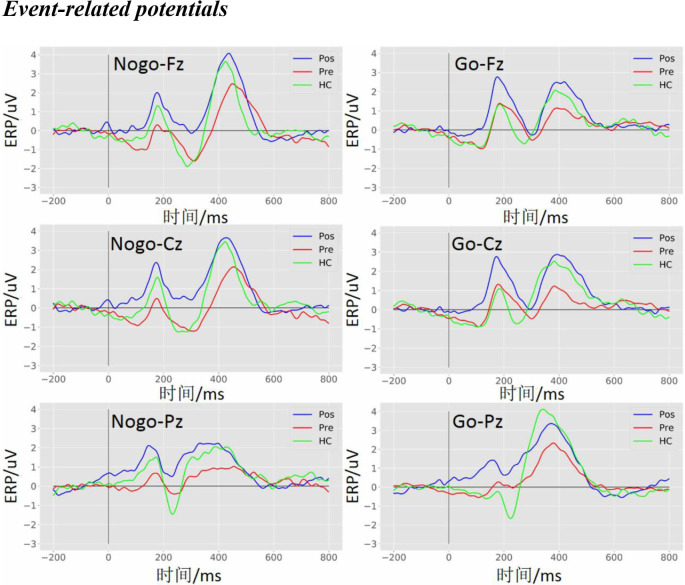
Event-related potentials (ERPs) waveform at the Fz, Cz, and Pz electrodes. The grand average ERPs waveform with three groups (pre, pre-operative group; post, post-operative group; HC, healthy control group) in the Nogo condition (left panel) and the Go condition (right panel) at the Fz, Cz, and Pz electrodes, respectively.

The ERPs elicited by the Go and the Nogo stimuli for the pre-operative group, post-operative group, and HCs are shown in Figure 3. The P300 amplitude (in the time window of 350–500 ms) was more positive in the post-operative group and HCs compared to the pre-operative group under the Go and the Nogo trials at the Fz, Cz, and Pz electrodes.

### The Amplitude of P300

**Figure 4:**
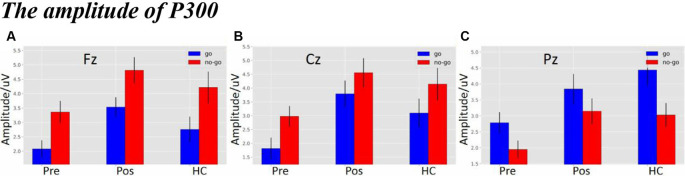
**(A)** The P300 amplitude among three groups under three different types of stimuli at Fz electrode. **(B)** The P300 amplitude among three groups under three different types of stimuli at Cz electrode. **(C)** The P300 amplitude among three groups under three different types of stimuli at Pz electrode (the time window of P300; pre, pre-operative group; post, post-operative group; HC, healthy control group).

As shown in Figure 4A, there was a significant difference in amplitude at the Fz electrode (*F*_(2,119)_ = 5.6629, *p* = 0.0045). The *post hoc* LSD test indicated that the post-operative P300 amplitude was significantly larger than that of the pre-operative group (*p* = 0.0010), while there were no differences between the HCs and the post-operative group (*p* = 0.1140) or between the HCs and the pre-operative group (*p* = 0.0791). Furthermore, a main effect of stimulus condition was obvious at the Fz electrode (*F*_(1,119)_ = 14.3752, *p* = 2.4114e-04). The *post hoc* LSD test indicated that the Nogo condition elicited an enhanced P300 amplitude compared to the Go condition (*p* = 0.0002). However, an interaction effect between stimulus condition and each group was not obvious at the Fz electrode (*F*_(2,119)_ = 2.8179e-02, *p* = 9.7222e-01).

As shown in Figure 4B, there was a significant difference in amplitude at the Cz electrode (*F*_(2,119)_ = 6.6983, *p* = 0.0017). The *post hoc* LSD test indicated that the post-operative P300 amplitude was significantly larger than that of the pre-operative group (*p* = 0.0005). The P300 amplitude of the HCs was significantly enhanced compared to that of the pre-operative group (*p* = 0.0153), while there was no difference between the HCs and the post-operative group (*p* = 0.2671). Additionally, a main effect of stimulus condition was obvious at the Cz electrode (*F*_(1,119)_ = 5.9316, *p* = 0.0016). The *post hoc* LSD test indicated that the P300 amplitude in the Nogo condition was significantly larger compared to the Go condition (*p* = 0.0164). However, an interaction effect between stimulus condition and each group was not seen at the Cz electrode (*F*_(2,119)_ = 8.6672e-02, *p* = 9.1703e-01).

As shown in Figure 4C, there was a significant difference in amplitude at the Pz electrode (*F*_(2,119)_ = 6.4523, *p* = 0.0022). The *post hoc* LSD test indicated that the post-operative P300 amplitude was significantly larger than that of the pre-operative group (*p* = 0.0064). The P300 amplitude of the HCs was significantly larger than that of the pre-operative group (*p* = 0.0010), while there was no difference between the HCs and the post-operative group (*p* = 0.5580). In addition, a main effect of stimulus condition was obvious at the Pz electrode (*F*_(1,119)_ = 8.7437, *p* = 0.0037). The *post hoc* LSD test indicated that the P300 amplitude in the Go condition was significantly larger than in the Nogo condition (*p* = 0.0164). However, an interaction effect between stimulus condition and group was not seen at the Pz electrode (*F*_(2,119)_ = 4.3350e-01, *p* = 6.4929e-01).

### The Latency of P300

**Figure 5:**
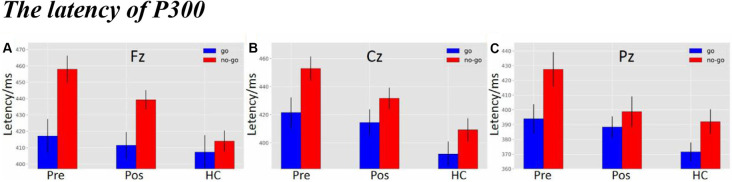
**(A)** The P300 latency among three groups under three different types of stimuli at Fz electrode. **(B)** The P300 latency among three groups under three different types of stimuli at Cz electrode. **(C)** The P300 latency among three groups under three different types of stimuli at Pz electrode (the time window of P300; pre, pre-operative group; post, post-operative group; HC, healthy control group).

As shown in Figure 5A, there was a significant difference in latency at the Fz electrode (*F*_(2,119)_ = 4.9138, *p* = 0.0089). The *post hoc* LSD test indicated that the pre-operative P300 latency was significantly longer than that of the HCs (*p* = 0.0022), while there were no differences between the pre-operative and the post-operative groups (*p* = 0.1597) or between the HCs and the post-operative group (*p* = 0.0890). Furthermore, a main effect of stimulus condition was obvious at the Fz electrode (*F*_(1,119)_ = 12.8705, *p* = 4.9311e-04). The *post hoc* LSD test indicated that the P300 latency in the Nogo condition was significantly longer than in the Go condition (*p* = 0.0004). However, an interaction effect between stimulus condition and group was not seen at the Fz electrode (*F*_(2,119)_ = 2.0132, *p* = 1.3827e-01).

As shown in Figure 5B, there was a significant difference in latency at the Cz electrode (*F*_(2,119)_ = 8.0522, *p* = 0.0053). The *post hoc* LSD test indicated that the pre-operative P300 latency was significantly longer than that of the HCs (*p* = 0.0001). The post-operative P300 latency was significantly longer than the HCs (*p* = 0.0162), while there was no difference between the pre-operative and the post-operative groups (*p* = 0.1261). Moreover, a main effect of stimulus condition was obvious at the Cz electrode (*F*_(1,119)_ = 8.5668, *p* = 0.0041). The *post hoc* LSD test indicated that the P300 latency in the Nogo condition was significantly longer than in the Go condition (*p* = 0.0041). However, an interaction effect between stimulus condition and group was not seen at the Cz electrode (*F*_(2,119)_ = 4.0119e-01, *p* = 6.7046e-01).

As shown in Figure 5C, there was a significant difference in latency at the Pz electrode (*F*_(2,119)_ = 4.7733, *p* = 0.0102). The *post hoc* LSD test indicated that the pre-operative P300 latency was significantly longer than that of the HCs (*p* = 0.0026), while there were no differences between the pre-operative and the post-operative groups (*p* = 0.2118) or between the HCs and the post-operative group (*p =* 0.0718). Furthermore, a main effect of stimulus condition was obvious at the Pz electrode (*F*_(1,119)_ = 7.7601, *p* = 0.0062). The *post hoc* LSD test indicated that the P300 latency in the Nogo condition was significantly longer than in the Go condition (*p* = 0.0062). However, an interaction effect of stimulus conditions and groups was not seen at the Pz electrode (*F*_(2,119)_ = 7.6012e-01, *p* = 4.6996e-01).

### The Topography of Average Amplitude in P300

**Figure 6:**
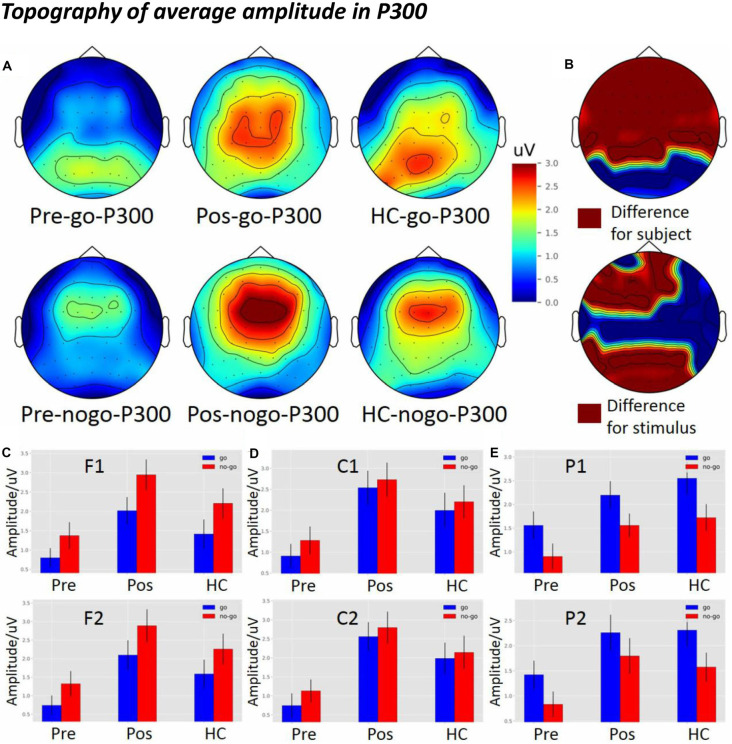
The average amplitude topography of P300 and the average amplitude of P300 among the three groups in Go and Nogo conditions.** (A)** The left, middle, and right columns show the topography of the pre-operative group, post-operative group, and HCs. The top and bottom panel indicate the topography of the Go and the Nogo trials, respectively. **(B)** The top panel shows the significant difference in topography among the subject groups, and the bottom panel shows the significant difference in topography among the different stimuli (*p* < 0.05) from two-way analysis of variance (ANOVA). **(C)** The P300 average amplitude among the three groups under different types of stimuli at the F1 and F2 electrodes. **(D)** The P300 average amplitude among the three groups under different types of stimuli at the C1 and C2 electrodes. **(E)** The P300 average amplitude among the three groups under different types of stimuli at the P1 and P2 electrodes. pre, pre-operative group; post, post-operative group; HC, healthy control group.

Topographical maps of ROIs (F1, F2, C1, C2, P1, and P2) and amplitude in the Go condition are depicted in Figure 6A (top panel). In the Go condition, P300 was distributed in the central and the posterior parietal cortex regions. P300 in the HCs and the post-operative group were more robust than in the pre-operative group.

Topographical maps of ROIs and amplitude in the Nogo condition are depicted in Figure 6A (bottom panel). In the Nogo condition, P300 was distributed in the frontal-central region. P300 in the HCs and the post-operative group were more robust than in the pre-operative group in the frontal-central region.

Figure 6B (top panel) shows significant differences between groups in the topography of average amplitude in the frontal, central, and parietal regions (*p* < 0.05). In the bottom panel, there were significant differences between stimulus conditions in the frontal and parietal regions (*p* < 0.05).

As shown in Figure 6C, there was a significant difference between the groups at the frontal electrodes (F1: *F*_(2,119)_ = 7.2759, *p* = 0.001; F2: *F*_(2,119)_ = 7.1291, *p* = 0.00120). The *post hoc* LSD test indicated that the post-operative P300 amplitude was significantly larger than that of the pre-operative group (F1: *p* = 0.0002; F2: *p* = 0.0002). The P300 amplitude of the HCs was significantly larger than that of the pre-operative group (F1: *p* = 0.0500; F2: *p* = 0.0239), while there was no difference between the HCs and the post-operative group (F1: *p* = 0.0693; F2: *p* = 0.1476). Furthermore, the main effect of stimulus condition was obvious at the frontal electrode (F1: *F*_(1,119)_ = 6.5600, *p* = 1.1734e-02; F2: *F*_(1,119)_ = 4.5852, *p* = 0.0343). The *post hoc* LSD test indicated that the P300 amplitude in the Nogo condition was significantly larger than in the Go condition (F1: *p* = 0.0117; F2: *p* = 0.0343). However, an interaction effect between stimulus condition and group was not seen at the frontal electrode (F1: *F*_(2,119)_ = 1.2033e-01, *p* = 8.8673e-01; F2: *F*_(2,119)_ = 3.6524e-02, *p* = 9.6414e-01).

As shown in Figure 6D, there was a significant difference between the groups at the central electrodes (C1: *F*_(2,119)_ = 8.0921, *p* = 0.0005; C2: *F*_(2,119)_ = 1.0059e-01, *p* = 9.4786e-05). The *post hoc* LSD test indicated that the post-operative P300 amplitude was significantly larger than that of the pre-operative group (C1: *p* = 0.0001; C2: *p* = 2.20e-05). The P300 amplitude of the HCs was significantly larger than that of the pre-operative group (C1: *p* = 0.0109; C2: *p* = 0.0050), while there was no difference between the HCs and the post-operative group (C1: *p* = 0.1715; C2: *p* = 0.1217). Furthermore, there was no main effect of stimulus condition at the central electrodes (C1: *F*_(1,119)_ = 6.5760e-01, *p* = 4.1909e-01; C2: *F*_(1,119)_ = 6.6033e-01; *p* = 4.1813e-01). In addition, an interaction effect between stimulus condition and group was not seen at the central electrodes (C1: *F*_(2,119)_ = 3.3269e-02, *p* = 9.6728e-01; C2: *F*_(2,119)_ = 4.3978e-02, *p* = 9.5699e-01).

As shown in Figure 6E, there was a significant difference between groups at the parietal electrodes (P1: *F*_(2,119)_ = 4.7980, *p* = 0.0099; P2: *F*_(2,119)_ = 4.8044, *p* = 0.0099). The *post hoc* LSD test indicated that the post-operative P300 amplitude was significantly larger than that of the pre-operative group (P1: *p* = 0.0339; P2: *p* = 0.0058). The P300 amplitude of the HCs was significantly larger than that of the pre-operative group (P1: *p* = 0.0032; P2: *p* = 0.0123), while there was no difference between the HCs and the post-operative group (P1: *p* = 0.3913; P2: *p* = 0.7898). Furthermore, a main effect of stimulus condition was obvious at the parietal electrodes (P1: *F*_(1,119)_ = 8.2845, *p* = 0.0047; P2: *F*_(1,119)_ = 5.1834, *p* = 0.0246). The *post hoc* LSD test indicated that the P300 amplitude in the Go condition was significantly larger than in the Nogo condition (P1: *p* = 0.0047; P2: *p* = 0.0246). However, an interaction effect of stimulus condition and groups was not seen at the Pz electrode (P1: *F*_(2,119)_ = 6.2621e-02, *p* = 9.3933e-01; P2: *F*_(2,119)_ = 9.0281e-02, *p* = 9.1373e-01).

We did not find any correlation between PRL levels and the amplitude of P300nogo (*p >* 0.05) or P300go (*p* > 0.05).

## Discussion

This study is the first to demonstrate impaired response activation and inhibition in prolactinoma patients. As expected, the patients showed longer reaction times and lower accuracy compared with the HCs. The present study also showed that P300nogo was larger than P300go in the frontal and central areas and that P300go was larger than P300nogo in the parietal region. The peak latency of P300 was significantly delayed in both pre- and post-operative groups compared to the HCs. Importantly, our findings reveal that there is a more prominent reduction of the P300 amplitude in the Nogo condition compared with the Go condition in prolactinoma patients. These patients also show improved amplitude of P300 in both Go and Nogo conditions at 6 months later after surgery, suggesting that: (1) there were likely deficits in response activation and inhibition in patients pre-operatively; and (2) improvements in response activation and inhibition could be seen after the surgical removal of the prolactin-secreting tumor. The results from the topographical maps provide fundamental evidence for the phenomenon of “anteriorization” of P300 in the Nogo condition compared to the Go condition.

Topographically, P300nogo was larger anteriorly compared with P300go. This is, to some extent, compatible with previous studies (Strik et al., 1998; Fallgatter and Strik, 1999). P300 in the Go condition is maximal at the centro-parietal sites, whereas P300nogo is maximal at the fronto-central sites (Kaiser et al., 2003; Smith et al., 2006). This phenomenon has been termed Nogo anteriorization (Fallgatter and Strik, 1999). This anteriorization of P300 in the Nogo condition may demonstrate response inhibition, which has been ascribed to an increase in the activity of frontal brain areas, particularly the ACC (Strik et al., 1998; Fallgatter et al., 2002; Enriquez-Geppert et al., 2010). Executive functioning is carried out by an extensive cerebral network which includes subcortical structures, thalamic pathways, and especially the PFC (Jurado and Rosselli, 2007). Executive functions typically include attention, inhibition, planning, problem-solving, working memory, and performance monitoring (Anderson et al., 2008). Neurological imaging studies have suggested that inhibitory mechanisms require the activation of the frontal lobe and other brain regions, such as PFC and ACC (Rubia et al., 2001). Additionally, it has been reported that response-inhibitory action particularly activates the frontal cortex (Munro et al., 2007). ERP source analysis has also revealed that P300nogo activity was observed in the frontal cortex (Bokura et al., 2001; Tian and Yao, 2008). In this study, we also found a larger fronto-central P300 in the Nogo condition and a larger parietal P300 in the Go condition.

Traditionally, the P300nogo amplitude is considered as a reflection of the motor-inhibitory neural substrate, and it demands more cognitive sources than the one required in classifying stimuli in certain conditions. Although the overlapping of movement-related activities may influence the difference between Go- and Nogo- ERPs within this time range (Falkenstein et al., 1999), the P300 modulation is generally considered to be an inhibitory mechanism. Therefore, its precise execution usually requires more cognitive resources and higher neural recruitment, which can be translated into higher amplitudes for the P300nogo component compared with P300go (Enriquez-Geppert et al., 2010; Gao et al., 2017). Our research suggests that the time interval of 350–500 ms after stimulus onset is late for information processing, and thus this interval likely reflects the response execution level. Therefore, a P300nogo effect evoked by motor-related areas reflects the response inhibition to this response execution attempt. This finding is in agreement with the concept that P300 reflects response inhibition (Bokura et al., 2001; Bruin and Wijers, 2002). The expected ERP morphology occurs in the HCs but is more variable in the prolactinoma patients, which may suggest alterations in information processing and task execution. Therefore, patients may need to recruit other resources to execute inhibitory processing, sustaining constant neural activation throughout the task.

The P300 amplitude decline is an obvious finding that is elicited with different paradigms in psychiatry. Patients with first-episode schizophrenia showed the decreased P300 in both Nogo and Go conditions (Kleinlogel et al., 2007), and patients with chronic schizophrenia manifested a decreased P300 only in the Nogo condition (Fallgatter et al., 2003). Previously, we found a slightly larger P300nogo in the HCs compared to the pre-operative group, but the peak latency of P300 was more delayed in the prolactinoma patients compared to the HCs (Cao et al., 2017). It is important to note that our previous study included patients with a prolactinoma, growth hormone-secreting pituitary tumors, and nonfunctional pituitary tumors. Here, we only recruited patients with prolactinomas and found significant P300 differences between groups in the frontal and central regions (see Figure 6B). The groups also showed a difference in latency. The longer latency of P300 in the pre-operative patients also suggests that both response activation and inhibition are significantly impaired at baseline by the prolactin-secreting tumor. Previous studies have shown that hormonal variation correlated with changes in specific brain structures (Peper et al., 2011), neuronal plasticity (Garcia-Segura and Melcangi, 2006), and functional compensation (Yao et al., 2020c). PRL excess can also impair cognition (Torner et al., 2013). Our team also revealed that gray matter volume decreases in the PFC of patients with prolactinomas (Yao et al., 2017), indicating that an abnormally high PRL level produces a negative effect on the brain structures related to inhibitory control. Interestingly, we also found that increased thalamocortical and cerebellar-cerebral functional connectivity was associated with endogenous hormone levels, which supports a functional compensatory mechanism that occurs before the cascade of structural damage (Yao et al., 2020c). PRL receptors (PRL-R) are widely expressed in the brain, including the thalamus, cerebral cortex, hypothalamus, and amygdala (Cabrera-Reyes et al., 2017). Therefore, patients with prolactinomas may have cognitive impairment due to damage in numerous brain structures. Interestingly, Bala et al. (2016) also speculated that PRL overproduction might influence the efficiency of cognitive functioning *via* the dopamine pathway, which could be altered in prolactinoma patients because of the anti-correlation between dopamine secretion and PRL production (Ben-Jonathan and Hnasko, 2001). In addition, PRL overproduction may be relevant in neuronal changes and plasticity in sensory systems, particularly in the brain cortex, because PRL significantly enhances the number of cells secreting antibodies that are directed against myelin oligodendrocyte glycoprotein (Correale et al., 2014). Oligodendrocytes are a type of neuroglia whose main function is to provide support and insulation to axons in the central nervous system (Ragheb, 1999). The PRL-R is distributed broadly in the brain and can mediate the regulation of neuronal functions, neuronal excitability, neurotransmission, and channels in the nervous system (Patil et al., 2014). Accordingly, the hypersecretion of these hormones can also directly affect brain structures that are relevant to cognitive functions. The present study shows the alterations in execution and inhibition in pre-operative patients, although we did not find any correlation between PRL levels and the amplitude of P300 in both Go and Nogo conditions. In line with previous research, Yedinak and Fleseriu (2014) further showed that the fluctuations in hormone levels do not uniformly correlate with the level of damage to cognitive functioning. Our previous findings suggest that patients with prolactinomas showed abnormal coherence between attention networks, but there were also no correlation between PRL levels and coherence among specific brain areas (unpublished data). This could be due to inadequate power because of the limited sample size. If the PRL is not high enough, it may not reach the threshold to elicit a correlation between PRL levels and coherence in attention networks.

Pre-operatively, there were significant alterations in response activation and inhibition in patients with a prolactinoma, showing decreased amplitude and delayed latency of P300. However, at 6 months after treatment, the post-operative patients showed improvement compared to the pre-operative patients. This suggests an improvement in the patients’ performance during the Go/Nogo task after tumor resection, which could be due to the normalization of hypothalamic–pituitary axes and secretory rhythms of pituitary hormones after the removal of the tumor. This is the first ERP study to show the positive effect of tumor removal on response activation and inhibition in prolactinoma patients.

### Limitations

Several limitations should be addressed. First, although there was a significantly decreased P300 amplitude and longer P300 latency in the pre-operative group, the relatively small sample is subject to selection bias and inadequate power, thus limiting generalizability. Second, the electrophysiological recordings are observed at the scalp level, so we cannot point out exactly which neuroanatomical regions are dysfunctional in these subjects. In the future, studies can use source analyses in conjunction with ERPs to further localize abnormal structures in the brain. Third, in the pre-operative patients, tumor size may have underlying effects on our results because studies have shown that the brain can undergo structural changes in patients with larger pituitary tumors (Guo et al., 2019; Rutland et al., 2019; Yao et al., 2020a). However, the study sample was strictly selected to exclude larger tumors that compress the optic apparatus or the surrounding brain structures. Therefore, we believe that the tumor size in our study may not bias our novel findings. Fourth, it should be noted that handedness does not uniformly determine hemispheric dominance. In a recent language task functional MRI (fMRI) study, Yao et al. (2020b) measured handedness in 36 patients and identified two patients (5.6%) who were right-handed and show right hemispheric dominance. Therefore, we suggest that hemispheric dominance and/or the strict requirements of handedness be taken into account in future studies. Moreover, we are seeing the application of various integrated neuroimaging methods such as structure MRI and resting-state fMRI (Catalino et al., 2020), which can map a more comprehensive cognitive and emotional network in neurosurgical patients and, potentially, enable the detection of both hemodynamic (blood-oxygen-level-dependent signal) and electrical sources of neural activity.

## Conclusion

For the first time, in patients with prolactinomas, we report a decrease of P300 amplitude and a longer P300 latency, which significantly improved in both Go and Nogo conditions after the patients achieved normal prolactin levels after surgical treatment. This suggests the recovery of response activation and inhibition in these patients after surgery. Therefore, P300 could be a potential biomarker of both impaired response activation and inhibition and recovery in patients with prolactinomas. The ERP–EEG technique could be used in clinical settings to quantitatively measure cognitive functioning due to its low cost, high temporal resolution, and efficiency.

## Data Availability Statement

The raw data supporting the conclusions of this article will be made available by the authors, without undue reservation, to any qualified researcher.

## Ethics Statement

The studies involving human participants were reviewed and approved by the ethics committee of Wuhan School of Clinical Medicine, Southern Medical University. The patients/participants provided their written informed consent to participate in this study.

## Author Contributions

LM and GX were responsible for the study concept and design. JS and CC contributed to the interpretation and the manuscript revision. DY collected the EEG data and revised the first draft of the manuscript with YW. JS and CC assisted with data analysis and interpretation of findings. JS, CC, and YW performed the statistical analysis. JS and CC drafted the manuscript. SY and MC critically revised the manuscript and assisted with English language editing. All the authors have reviewed the content and approved the final version for publication.

## Conflict of Interest

The authors declare that the research was conducted in the absence of any commercial or financial relationships that could be construed as a potential conflict of interest.
